# Mild hypothermia improves brain injury in rats with intracerebral hemorrhage by inhibiting IRAK2/NF‐κB signaling pathway

**DOI:** 10.1002/brb3.1947

**Published:** 2020-12-14

**Authors:** Hui Shi, Zulu Su, Hai Su, Hao Chen, Yi Zhang, Yuan Cheng

**Affiliations:** ^1^ Department of Neurosurgery YongChuan Hospital Chongqing Medical University Chongqing China; ^2^ Department of Neurosurgery The Second Affiliated Hospital of Chongqing Medical University Chongqing China

**Keywords:** apoptosis, brain injury, intracerebral hemorrhage, IRAK2/NF‐κB pathway, mild hypothermia

## Abstract

**Objective:**

To explore the effect of mild hypothermia on nerve injury by establishing a rat model of intracerebral hemorrhage (ICH), and to clarify the specific molecular mechanism of mild hypothermia in improving brain injury in ICH rats.

**Methods:**

The rat model of ICH was established by collagenase injection. The neurological deficit score (NDS), brain tissue water detection, and Nissl staining were applied to detect the degree of brain injury. ELISA was used to analyze the expression of proinflammatory cytokines and serum nerve injury indexes. Flow cytometry and Western Blot were used to detect neuronal apoptosis.

**Results:**

Mild hypothermia treatment significantly improved the brain injury of the ICH rats and down‐regulated the inflammatory response and oxidative stress in the brain tissue. Moreover, mild hypothermia also effectively inhibited IRAK2/NF‐κB signaling pathway and thus affect neuronal apoptosis.

**Conclusion:**

Mild hypothermia alleviates inflammatory response and neuronal apoptosis by inhibiting IRAK2/NF‐κB signaling pathway in the ICH rats thus improving brain injury.

## Graphical abstract

Mild hypothermia alleviates brain tissue inflammation and neuronal apoptosis by inhibiting RAK2/NF‐κB signal pathway in rats with intracerebral hemorrhage, thus improving brain injury.

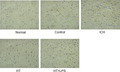

## 1 INTRODUCTION

Intracerebral hemorrhage (ICH) refers to the hemorrhage caused by nontraumatic rupture of microvascular vessel in brain parenchyma, and it is a cerebrovascular disease with high mortality rate (Michinaga et al., 2014). Severe ICH leads to loss of nervous system function in newborns, and the mortality rate of acute ICH in adults is as high as 40% (Praveen, 2010; Sprigg et al., 2018). Intracerebral hemorrhage causes hematoma and neuronal necrosis or apoptosis in the brain tissue around the hematoma, and results in inflammatory response and oxidative stress in the brain tissue (Haiping et al., 2016; King et al., 2014; Wang et al., 2020). Targeted antioxidant therapy can effectively protect brain tissue and alleviate brain injury (Liu et al., 2019). However, the existing treatment for ICH includes hematoma clearance and symptomatic treatment, and there is no more effective treatment currently (Keep et al., 2012, 2018). Hematoma clearance cannot effectively improve the prognosis of patients, but the secondary injury of ICH can be alleviated, thus significantly improving the symptoms after ICH (Hu et al., 2019; Lai et al., 2019). Therefore, clinically, a more perfect treatment plan is needed to ensure a good prognosis of ICH patients.

Recent studies have shown that mild hypothermia adjuvant therapy has been widely used in clinical research and clinical treatment of a variety of brain diseases, such as neonatal hypoxic‐ischemic encephalopathy, severe craniocerebral injury (Ifadah et al., 2020; Lijun et al., 2020). Mild hypothermia plays a positive role in the treatment of hypoxic‐ischemic encephalopathy (Chawla et al., 2020; Lijun et al., 2020) and can protect the body from a variety of cellular pressure. It has been reported that mild hypothermia treatment at 33°C can reduce the oxidative stress damage of PC12 cell lines induced by peroxide (Jayanti et al., 2020). Mild hypothermia improves reperfusion injury and its poor prognosis in ischemic stroke and plays a neuroprotective role (Hassanipour et al., 2020). However, the specific mechanism of mild hypothermia in improving brain injury after ICH is not clear.

The interleukin‐1 receptor‐associated kinases (IRAKs) in the human genome, including IRAK1, IRAK2, IRAK4, and IRAKM, are key mediators of the TLR/41L‐1R (TIR) signaling pathway (Sridevi et al., 2008; Keating et al., 2007; Tan et al., 2008). TLR/IL‐1R‐mediated NF‐κB activation requires all IRAK proteins, except IRAKM. Studies have found that compared with IRAK1, IRAK2 plays a more important role in TLR‐induced NF‐κB activation. Expression of IRAK2 can lead to TRAF6 ubiquitination, which is a key event in NF‐κB activation (Keating et al., 2007). In addition, the interaction of IRAK2‐TRAF6 is necessary to maintain IkappaB kinase (IKK) activity during prolonged activation of MyD88 signaling (Eduardo et al., 2013). Mild hypothermia, on the other hand, can inhibit the phosphorylation of inhibitor protein of NF‐κB (IkappaB‐alpha) by decreasing the expression and activity of IKK, thus exerting a protective effect on brain injury (Yenari & Han, 2006). It has been confirmed that IRAK4 is highly expressed in microglia of mice with ICH (Yuan et al., 2015), and increasing the expression of IRAK1 may lead to the expansion of ICH (Jia et al., 2010). Cui et al (Guo et al., 2010) have shown that IRAK2 expression is higher in the brains of elderly patients with Alzheimer's disease. However, the study of IRAK2 in ICH has not been reported.

Therefore, in this study, a rat model of ICH was constructed by collagenase injection, and then treated with mild hypothermia and Lipopolysaccharide (LPS), so as to explore the effect of mild hypothermia on brain injury in rats with ICH. Our results showed that mild hypothermia treatment could improve brain injury by inhibiting IRAK2/NF‐κB signaling pathway, thus reducing neuronal injury and inflammatory response in brain tissue of rats with ICH.

## 2 MATERIALS AND METHODS

### 2.1 Ethics statement

All the animal experiments in this study were approved by the YongChuan Hospital, Chongqing Medical University Ethics Committee. The animal experiments were carried out under the conditions of abiding by the Animal Protection Law of the people's Republic of China and the rules.

### 2.2 Establishment of animal model

Fifteen 8‐week‐old *SD* male rats (weight: 250–300 g) were randomly divided into five groups, including normal group, sham operation group (control), intracerebral hemorrhage model (ICH) group, mild hypothermia (HT) group, and mild hypothermia + Lipopolysaccharide (HT + LPS) group with 3 rats in each group. The rats in the normal group were fed in an incubator at 25 ℃ without any treatment. The construction of ICH rat model mainly refers to the method previously described by Guo et al. (2016). The rats were anesthetized with 10% 0.3 g/L chloral hydrate (Sigma), and then fixed on the brain stereotaxic instrument (Leica). A sagittal incision was made in the middle of the eyes and ears of the rats; a burr hole was opened at 0.2 mm posterior to the bregma and 3 mm to the right of the sagittal line, and 1 μL 0.25 U type IV collagenase (Sigma) was injected into the hole. During the operation, the rectal temperature was kept at 37‐38°C, and the rats were cultured in an incubator at 25°C for 24 hr after operation. In the control group, type IV collagenase was not injected into the rats, and the other procedures were the same as those in the ICH group.

The treatment of mild hypothermia was according to the study of Guo et al. (2016). In brief, a glass box of 80 cm × 60 cm × 45 cm was prepared with crushed ice filled between the inner box and the outer box, and the outer box was wrapped with foam plastic for insulation. A thermometer was placed in the inner box, and the temperature was maintained at approximately 5°C. The ICH model rats were cultured in an incubator at 25°C for 12 hr and then placed in the inner box. The rats were taken out every 20 min to rapidly measure their rectal temperature using a digital thermometer, and then put back. After the rectal temperature was below 35℃, the rectal temperature was measured every 30 min to maintain it at (33 ± 1) °C. After it was lower than 32°C, the rats were taken out, warmed to 34°C at room temperature and then put back into the box. The above procedures lasted 12 hr. In the HT + LPS group, 5 mg/kg LPS (Solarbio, China) was injected intraperitoneally before the ICH model was constructed, and the other procedures were the same as those in the HT group. The rats in each group were anesthetized with chloral hydrate after 24 hr of treatment. Subsequently, the chest was open, and apical intubation was performed through left ventricular to aorta. The right atrial appendage was cut open, and 100 ml of normal saline was rapidly perfused. After the liver became uniform pink, 250 ml of 4% paraformaldehyde was dripped, first quickly and then slowly, which was completed in 1 hr. Next, the rats were decapitated and the brains were removed and fixed overnight in 4% paraformaldehyde. The incision was made along the coronal plane of the injection site; 3 mm thick brain tissues were collected and sent to the Pathology Laboratory of Xuzhou Medical College for HE staining, while 3 mm thick brain tissues was immersed in 30% sucrose for dehydration for 4–6 hr and embedded using a cryostat to make frozen sections.

### 2.3 Neurological deficit score

After 24 hr of treatment, the NDS of the rats in each group was assessed following the method previously described by Peeling (2001). NDS items included spontaneous circling (Graded 0 for a rat does not circle to 3 for a rat continues to circle), hindlimb retraction (Pull the rat's lower limbs out by 2–3 cm. Graded 0 for a rat retracts to the original position immediately and graded 3 for a rat retracts to the original position after a few seconds or no retraction), bilateral forepaw grip (Both forelimbs of the rat are hung on a steel wire rope with a diameter of 2 mm. Graded 0 for a rat is waiting for >5 s, and graded 3 for a rat falls from the rope at 0–2 s), bilateral forelimb flexion (Lift the tail of the rat to observe the extension of both forelimbs. Graded 0 for a rat's forelimbs are fully extended, and graded 3 for forelimb flexion) and walk measurement (Ability to move along a 70 cm × 2.4 cm‐wide wood beam. Graded 0 for a rat passed smoothly to 3 for a rat unable to stay). The score of each item is divided into different scores according to the severity; 3 is the highest score, and the upper limit of the total score is 15. Higher score indicates more severe neurological deficits.

### 2.4 Brain tissue water detection

The tissues around the hematoma were washed, dried the surface water and weighed (W1). After weighing, the tissues were dried in an oven at 60 ℃, and weighed after about 48 hr (W2). Water content rate of brain tissue Water rate = (W1–W2) ÷ W2 × 100% = (W1 − W2) ÷ W2 × 100%.

### 2.5 Nissl staining

Nissl staining was used to determine the neuronal injury in the brain tissue around the hematoma. The sections were fixed in 4% paraformaldehyde for 10 min, rinsed with distilled water and then stained with Nissl staining solution (Beyotime) for 5 min. Subsequently, the sections were rinsed with distilled water twice followed by dehydration with 95% ethanol and cleared with xylene. Finally, the sections were mounted with neutral resin.

### 2.6 Immunohistochemistry

The sections were incubated with 0.5% TritonX‐100 for 10 min and sealed with goat serum at room temperature for 10 min. Then the sections were incubated with the FITC‐labeled secondary antibody (SouthernBiotech) in the dark at room temperature, and finally covered with 50% glycerol on the slide. The image was obtained using a fluorescence inverted microscope (Leica).

### 2.7 Flow cytometry

The preparation of single‐cell suspension was as follows. After the rats in each group were sacrificed, the brain tissues were quickly separated on ice. About 30 mm^3^ brain tissues around the hematoma were cut using an ophthalmic scissor and then cut into pieces in a culture dish containing 1 ml of PBS. The tissues were added 50 μl of 0.25% trypsin, mashed several times using a tube, and then filtered through a 200‐mesh filter into a centrifuge. PBS was added to dilute to 5 ml. Next, and the centrifugation was carried out at 1,000 rpm for 15 min. After that, the tissues were rinsed twice with PBS, fixed with ethanol for 3 hr. The number of cells was 1 × 10^6^/ml. The detection of cell apoptosis was performed using Attune NxT flow cytometry (ThermoFisher), and in accordance with the operation instructions of Annexin V‐FITC/PI cell apoptosis kit (Beyotime).

### 2.8 Enzyme‐linked immunosorbent assay (ELISA)

The serum of rats was collected and stored at −20°C. With the serum as samples, the contents of serum cytokines TNF‐α, IL‐6, IL‐8, IL‐1β and nerve injury indexes MBP, Smur100B, and NSE were detected according to the instructions of ELISA kit (mlbio). ELISA reader (BioTek) was applied to analyze the results.

### 2.9 Analysis of the contents of superoxide dismutase (SOD), glutathione peroxidase (GSH‐Px), and malondialdehyde (MDA)

With the rat serum as samples, ELISA reader (BioTek) was applied to analyze the contents of SOD, GSH‐PxX, and MDA. The procedures were operated according to the instructions of Total Superoxide Dismutase Assay Kit (Beyotime), Glutathione Peroxidase Assay Kit (Beyotime), and Lipid Peroxidation MDA Assay Kit (Beyotime).

### 2.10 Western blot

The denatured proteins were separated with 12% SDS‐PAGE (Beyotime, China). The electrophoretic conditions were 100V for 20 min and 120 V for 60 min. The protein was transferred to PDVF membrane by wet transfer method (BioRad). The membrane was incubated with the first antibody, and then secondary antibody (ZenBio) was used for protein labeling. The protein bands were developed using the color‐substrate solution (Beyotime) and photographed in a two‐color infrared laser imaging system (Li‐cor).

### 2.11 Statistical analysis

All the results in this study were expressed as mean ± standard deviation (*SD*), and all the experiments were repeated three times independently. The differences between groups were analyzed by two‐tail *t* test or one‐way analysis of variance (ANOVA). *p* < .05 were considered significant.

## 3 RESULTS

### 3.1 Mild hypothermia improves brain injury in rats with ICH

**Figure 1:**
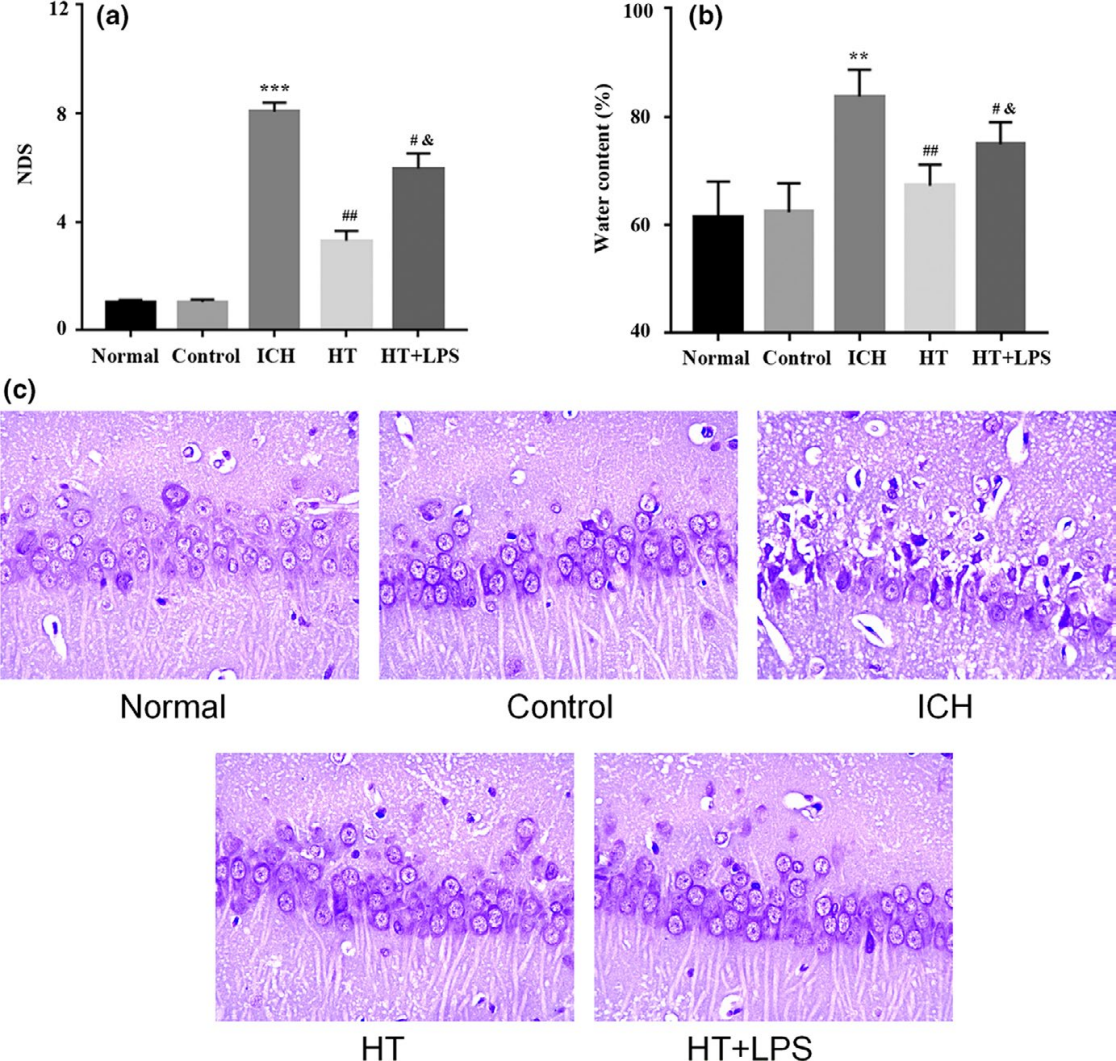
Mild hypothermia improves brain injury in rats with intracerebral hemorrhage. (a) Neurological deficit score of rats. (b)The water content of the brain tissue around the hematoma. (c) Detection of neuronal injury by Nissl staining. **p < .01 and ***p < .001 versus control group, ^#^ *p* < .05 and ^##^ *p* < .01 versus ICH group, ^&^ *p* < .05 versus HT group. Control, sham operation group; ICH, intracerebral hemorrhage model group; HT, mild hypothermia group; HT + LPS, mild hypothermia + Lipopolysaccharide group. NDS, Neurological deficit score

In order to directly observe the effects of different treatments on rats with ICH, NDS was used to evaluate brain injury. The results of NDS showed that the NDS of the rats in the ICH group was significantly higher than that of control group. However, the NDS in the HT group and HT + LPS group was significantly lower than that in the ICH group, and the treatment of LPS increased the NDS of ICH rats after hypothermia treatment (Figure 1a). In addition, the water content of the brain tissue adjacent to the cerebral hematoma was also detected. The results (Figure 1b) showed that the water content of brain tissue in the ICH group was significantly increased compared with that in the control group. After mild hypothermia treatment, the water content of brain tissue in ICH rats was significantly decreased. Compared with the HT group, the brain water content of the HT + LPS group was increased. Nissl staining was used to further evaluate the effects of mild hypothermia treatment on brain nerve injury in ICH rats. As shown in Figure 1c, there was obvious cytoplasmic atrophy of neurons in the ICH group. However, the cytoplasmic atrophy of neurons in the HT group and HT + LPS group was less than that in the ICH group. Compared with the HT group, the cytoplasmic atrophy of neurons in the HT + LPS group was more significant. These results suggested that mild hypothermia could effectively improve the brain injury in rats with ICH.

### 3.2 Mild hypothermia down‐regulates the protein expression of IRAK2 in brain injury

**Figure 2:**
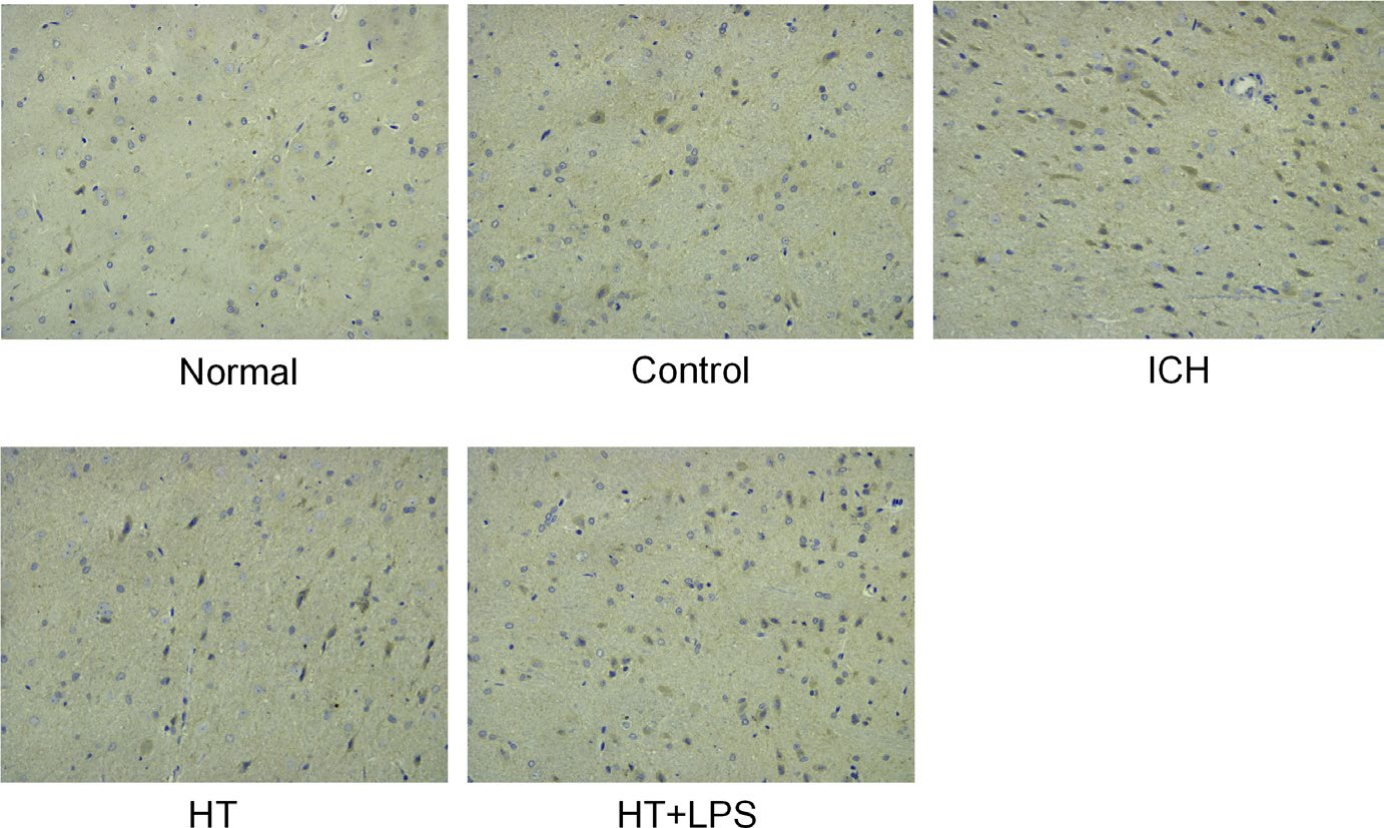
Mild hypothermia down‐regulates the protein expression of IRAK2 in brain injury. Immunohistochemistry was used to detect the expression of IRAK2 in the brain tissue around the hematoma in each group

IRAK2 immunohistochemical experiment was carried out on the brain tissue around cerebral hematoma in the ICH rats. The results showed that compared with the control group, the expression of IRAK2 in the brain tissue of the ICH group was significantly increased, while the expression of IRAK2 in brain tissue of the HT group and HT + LPS group was significantly lower than that of the ICH group. Compared with the HT group, the IRAK2 expression in HT + LPS group was significantly higher in the HT group (Figure 2). These results showed that the expression of IRAK2 was up‐regulated in the brain injury of the ICH rats; mild hypothermia treatment could effectively inhibit this up‐regulation, but the stimulation of LPS could increase the expression of IRAK2.

### 3.3 Mild hypothermia inhibits neuronal apoptosis and reduces inflammation caused by ICH

**Figure 3:**
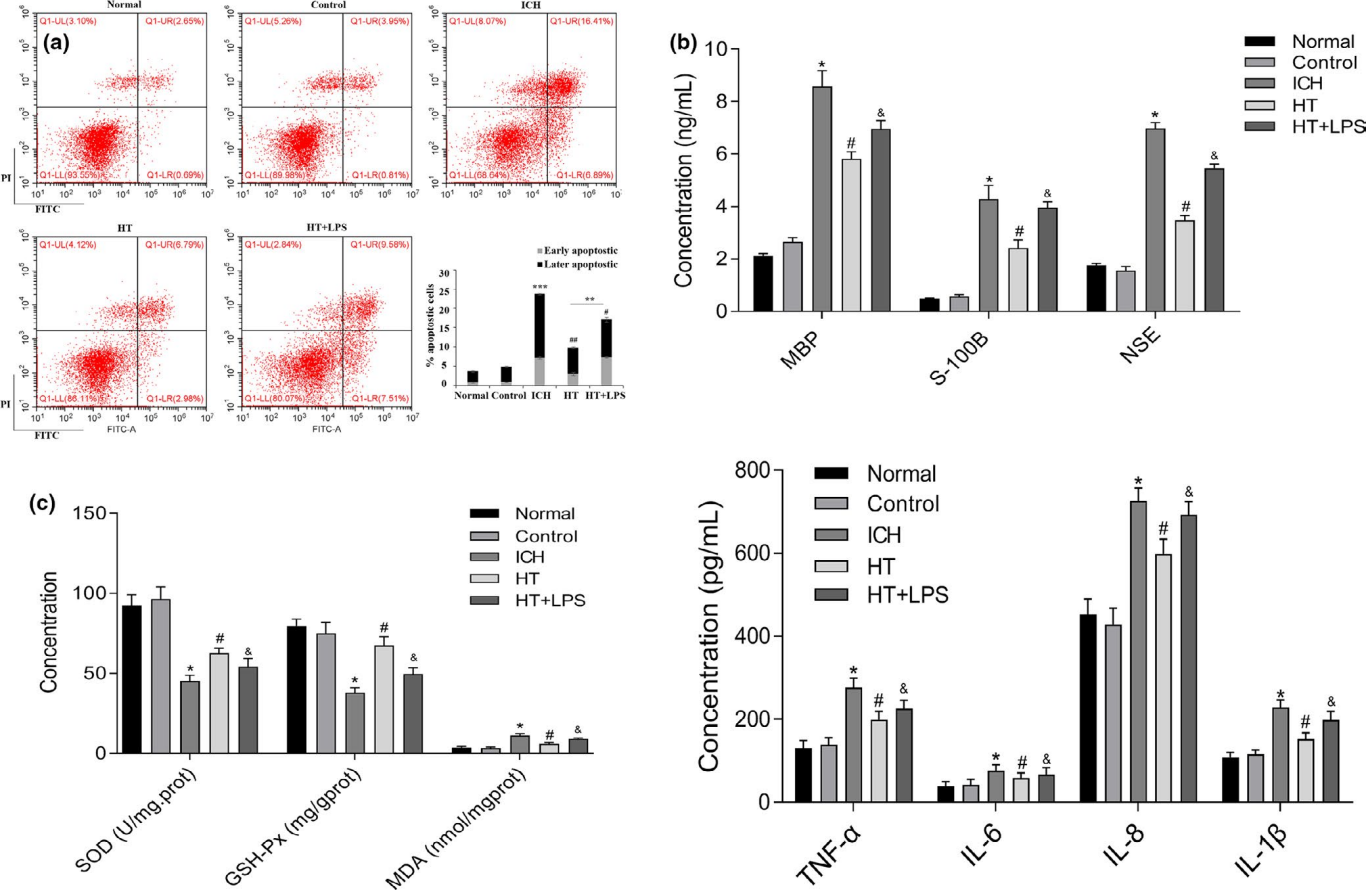
Mild hypothermia inhibits neuronal apoptosis and reduces inflammatory response caused by intracerebral hemorrhage. (a) Detection of neuronal apoptosis by flow cytometry. (b) Detection the content of MBP, S‐100B, and NSE in serum of rats by ELISA. (c) Expression levels of GSH‐Px, MDA, and SOD in brain tissue around hematoma. (d) Detection of TNF‐α, IL‐6, IL‐8, and IL‐1β in rat serum by ELISA. The results are expressed as Mean ± *SD*. *p < .05 versus control group,, ^#^ *p* < .05 versus ICH group, ^&^ *p* < .05 versus HT group

Based on the condition of nerve cell injury in ICH rats, the apoptosis rate of neurons in the tissue around the hematoma was detected by flow cytometry. The results showed (Figure 3a) that the neuronal apoptosis rate in the brain tissue of the ICH rats, while after mild hypothermia treatment, the neuronal apoptosis rate of the ICH rats was significantly reduced. Compared with the HT group, the apoptosis rate in the HT + LPS group was significantly higher. ELISA was further used to detect the content of nerve injury indexes MBP, S‐100B, and NSE in rat serum. The results showed that MBP, S‐100B, and NSE in the ICH group were increased significantly. After mild hypothermia treatment, the expression of these indicators was significantly reduced, and the expression in the HT + LPS group was higher than that in the HT group (Figure 3b). ICH can increase ROS, causing cell damage in the form of lipid peroxidation (Yu‐shu et al., 2014). In order to explore whether the neuroprotective mechanism of mild hypothermia is related to its antioxidant capacity, the expression levels of GSH‐Px, SOD, and MDA in the brain tissue were detected. The results showed that mild hypothermia treatment could effectively increase the expressions of GSH‐Px and SOD and decreases MDA expression in the ICH rats, while LPS stimulation reduced the effects of mild hypothermia on GSH‐Px, MDA, and SOD (Figure 3c).

Finally, the expression of inflammatory factors was detected by ELISA to further confirm the anti‐inflammatory effect of mild hypothermia on ICH rats. Figure 3d showed that the expression of TNF‐α, IL‐6, IL‐8, and IL‐1β in the serum of ICH rats was significantly up‐regulated. Mild hypothermia could effectively reduce the expression of proinflammatory cytokines, while LPS could reverse the inhibitory effect of mild hypothermia on the expression of proinflammatory cytokines.

These results confirmed that mild hypothermia could effectively inhibit the apoptosis of neurons and reduce the inflammatory response and oxidative stress in the brain tissue of ICH rats.

### 3.4 Mild hypothermia inhibits IRAK2/NF‐κB signaling pathway and neuronal apoptosis

**Figure 4:**
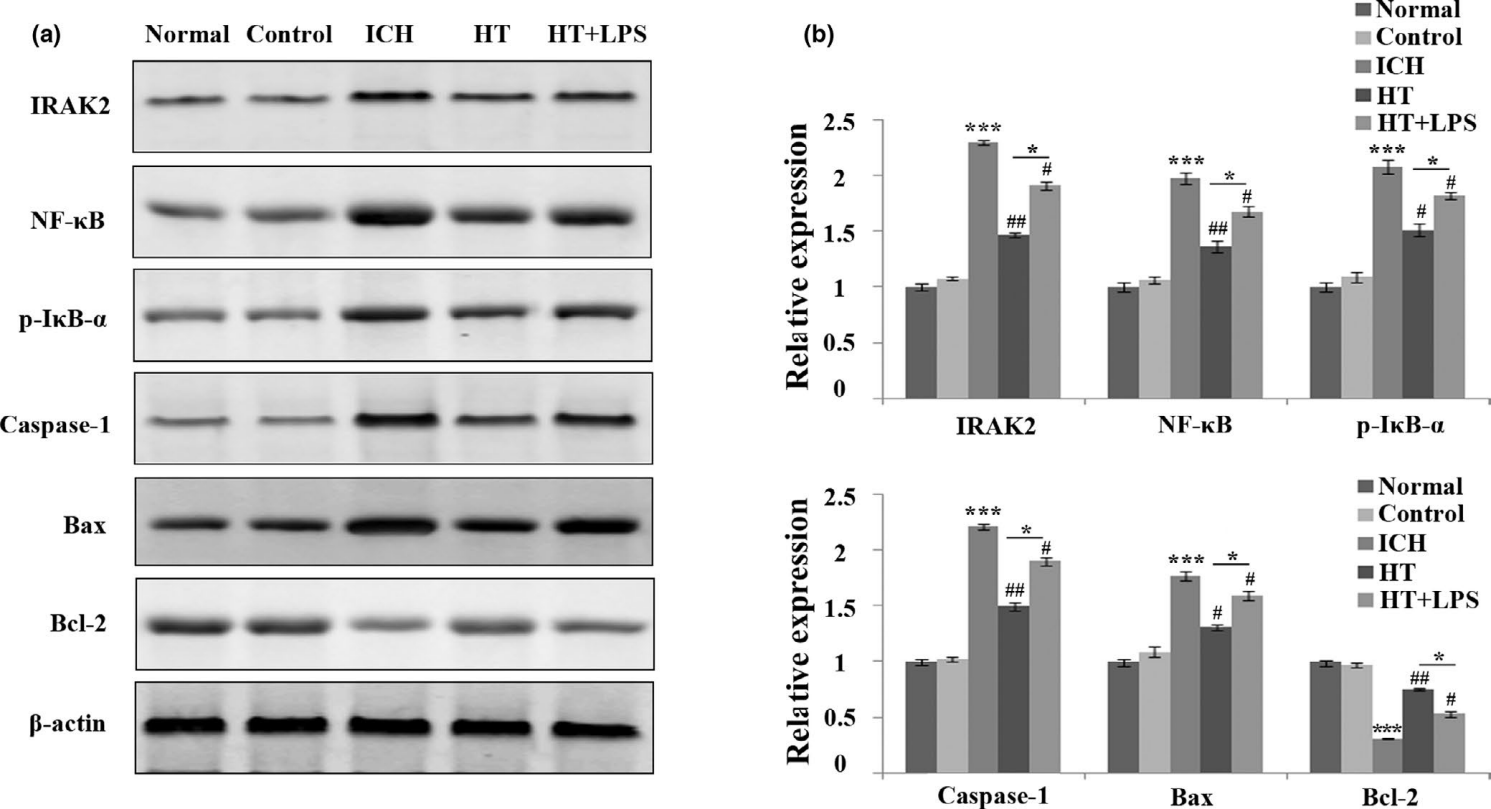
Mild hypothermia inhibits IRAK2/NF‐κB signal pathway and inhibits neuronal apoptosis. (a) The expression of IRAK2, NF‐κB, p‐IκB‐α, caspase‐1, Bax, and Bcl‐2 is measured by Western blot. (b) Grayscale analysis of protein bands. The results are expressed as Mean ± *SD*. *p < .05 and ***p < 0.001 versus control group, ^#^ *p* < .05 and ^##^ *p* < .01 versus ICH group

The results of immunohistochemistry have proved that mild hypothermia reduced the protein expression of IRAK2 in brain tissue of ICH rats. Therefore, Western blot was used to further clarify whether mild hypothermia could improve brain injury through IRAK2/NF‐ κB signaling pathway. The results (Figure 4) showed that the protein expressions of IRAK2, NF‐κB, p‐IκB‐α, caspase‐1, and Bax in the brain tissue of ICH rats were increased, while the expression of Bcl‐2 was decreased. However, mild hypothermia treatment could significantly decrease the protein expression of IRAK2, NF‐κB, p‐IκB‐α, caspase‐1, Bax while increase the protein expression of Bcl‐2 in brain tissue of ICH rats; LPS stimulation could reverse the expression of the above‐mentioned proteins in the HT group. These results proved that mild hypothermia could effectively inhibit IRAK2/NF‐κB signaling pathway in ICH rats and thus inhibit neuronal apoptosis.

## 4 DISCUSSION

ICH, as a clinical critical disease with a high mortality rate, seriously affects people's health and life (Krishnamurthi et al., 2013). At present, its major clinical treatment such as hematoma clearance, cannot improve the prognosis of patients (Mendelow et al., 2005, 2013). The secondary injuries including inflammatory response, oxidative stress, and apoptosis are the main causes of neurological dysfunction in ICH patients after treatment (Haiping et al., 2016). In this study, we found that mild hypothermia could effectively improve the brain injury of ICH reduce the apoptosis of neurons in the brain tissue around the hematoma, alleviate the inflammatory response of brain tissue and relieve oxidative stress. More importantly, in this study, we found that mild hypothermia improved brain injury in ICH rats by inhibiting the IRKA2/NF‐κB signaling pathway.

Clinically, ICH will cause neurological dysfunction in patients (Haiping et al., 2016; King et al., 2014). In this study, the rat model of ICH was established by collagenase injection. The NDS of ICH rats was significantly higher than that of the control group. There were obvious neurological deficits in the ICH rats. Nissl staining showed the atrophy of neurons in the brain tissue of ICH rats, and the serum nerve injury indexes also confirmed the nerve injury in the ICH rats. However, the treatment of mild hypothermia significantly reduced the neurological deficit and effectively improved the nerve injury in the ICH rats.

Mild hypothermia has been proved to improve neuronal injury in ICH rats by inhibiting neuronal apoptosis induced by endoplasmic reticulum reaction (Guo et al., 2016). Studies have shown that ICH can cause neuronal necrosis, apoptosis, and autophagy in the brain tissue around the hematoma (King et al., 2014; Lotocki et al., 2006). This is consistent with our results. Our study also confirmed the cytoplasmic atrophy of neurons in the brain tissue of the ICH rats. Flow cytometry also showed that the neuronal apoptosis rate in the brain tissues of the ICH rats was significantly higher than that in the control group, and mild hypothermia treatment could significantly reduce the neuronal apoptosis, reduce the protein expressions of caspase‐1 and Bax, while increase the protein expression of Bcl‐2.

In addition, we found that LPS stimulation could significantly attenuate the improvement effect of mild hypothermia on nerve injury in the ICH rats, which implied that mild hypothermia also reduced the inflammatory response caused by ICH. It has been reported that the severity of ICH is related to proinflammatory cytokines in serum (Wu et al., 2014). Inflammatory cytokines can lead to the infiltration of peripheral inflammatory cells and thus damage neurons (K et al., 2013). Reducing the expression level of inflammatory cytokines in serum can effectively inhibit the inflammatory response after ICH, thus reducing brain injury (Christopher et al., 2003). Our study showed that mild hypothermia reduced serum inflammatory cytokines in the ICH rats, indicating that mild hypothermia improved brain injury in ICH rats by reducing the expression of inflammatory cytokines. In addition, mild hypothermia could reduce the expression of IRKA2 in the brain tissue of ICH rats, and IRKA2 has been proved to promote the inflammatory response of the body (Shuai et al., 2019). This result implied that mild hypothermia may improve the inflammatory response in rats with ICH by regulating the expression of IRKA2. NF‐κB signaling pathway has been reported to activate in ICH and aggravate the secondary injury after ICH (Liu et al., 2016). However, IRKA, as the upstream regulatory factor of NF‐κB, indicates that mild hypothermia may alleviate the inflammatory response of brain tissue in rats with ICH through IRKA2/NF‐κB signaling pathway. Mild hypothermia could reduce the protein expression levels of IRKA2, NF‐κB, and p‐IκB‐α, which confirmed that mild hypothermia inhibited the IRKA2/NF‐κB signaling pathway and weaken the inflammatory response in the brain tissue of rats with ICH, thus improving the brain injury in ICH.

## 5 CONCLUSION

In summary, mild hypothermia could effectively inhibit IRKA2/NF‐κB signal pathway, and then inhibit neuronal apoptosis and weaken the inflammatory response in the brain tissue, thus improving the brain injury in ICH rats.

## ETHICS APPROVAL AND CONSENT TO PARTICIPATE

Not Applicable.

## CONSENT FOR PUBLICATION

Not Applicable.

## Conflict of interest

The authors claim that there is no conflict of interest between them.

## AUTHOR CONTRIBUTIONS

H Shi and Y Cheng involved in study concept and design, drafting of the manuscript, critical revision of the manuscript for important intellectual content, and statistical analysis. Z Su and H Su involved in acquisition of data. H Chen and Y Zhang involved in analysis and interpretation of data. All authors have read and approved the manuscript.

### Peer Review

The peer review history for this article is available at https://publons.com/publon/10.1002/brb3.1947.

## Data Availability

Availability of data and materials: Data available in Article.
